# Supplementary material for: Dendrogenin A Synergizes with Cytarabine to Kill Acute Myeloid Leukemia Cells In Vitro and In Vivo
Source: Cancers (Basel). 2020 Jun 29;12(7):1725. doi: 10.3390/cancers12071725 (PMC7407291; doi:10.3390/cancers12071725)
Supplement: Supplementary file 1 [file cancers-12-01725-s001.pdf]

Supplementary Materials: Dendrogenin A synergizes with Cytarabine to Kill Acute Myeloid Leukemia Cells In Vitro and In Vivo

Nizar Serhan, Pierre-Luc Mouchel, Philippe de Medina, Gregory Segala, Aurélie Mougel, Estelle Saland, Arnaud Rives, Antonin Lamaziere, Gaëtan Despres, Jean-Emmanuel Sarry, Clément Larrue, François Vergez, Laetitia Largeaud, Michel Record, Christian Récher, Sandrine Silvente-Poirot and Marc Poirot

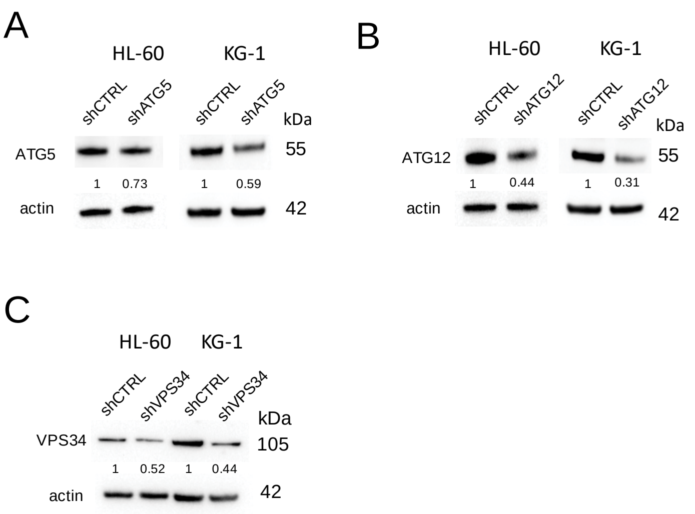

Figure S1. Immunoblot for ATG5, ATG12 and VPS34 expression in HL6-60 and KG-1 cells transfected with sh control (shCTRL) or sh against ATG5 (shATG5), ATG12 (shATG12) or VPS34 (shVPS34).

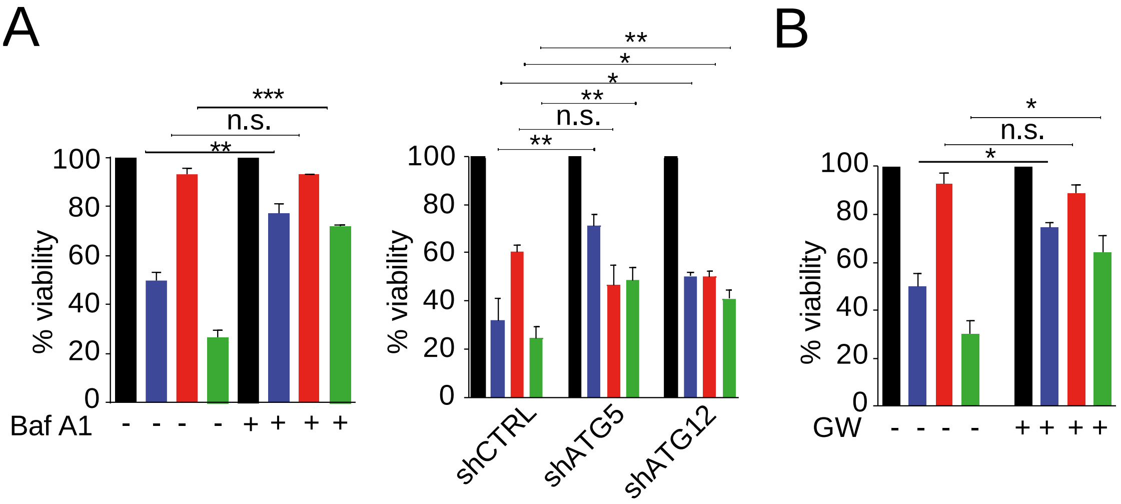

Figure S2. Effect of pharmacological and genetic inhibition of autophagy and of the LXRβ agonist GW3965 on DDA potentiation of Ara-C cytotoxicity. MV4-11 cells were treated for 48h with 5μM DDA and 0.1μM Ara-C, alone or in combination, in the absence or presence of (A) 5 nM bafilomycin A1 (Baf A1). (B) MV4-11 cells transfected with control shRNA (shCTRL) or shRNA against ATG5 (shATG5) and against ATG12 (shATG12) were treated for 48h with 5μM DDA and 0.1μM Ara-C, alone or in combination. (C) MV4-11 cells were treated for 48h with 5μM DDA and 0.1μM Ara-C, alone or in combination, in the absence or presence of 1 μM of GW 3965. Viability was measured as described above. Bars are mean ± SEM of five independent experiments. * $p < 0.05$, ** $p < 0.01$, *** $p < 0.001$.

Table S1. Molecular and cytogenetic characteristics of primary AML cancer cells from 20 patients.

Patient	Karyotype	Pronostic	FLT3-ITD	FLT3-TKD	NPM1	DEBA	IDH1	IDH2	DNMT3A
#**AML 1**	46,XY<20>	IR	+	-	+	-	-	-	+
#**AML 2**	normal	IR	+	-	+	-	-	-	-
#**AML 3**	normal	IR	-	+	+	-	-	-	-
#**AML 4**	46,XX,del(20)(q11q13)<20>	IR	-	-	-	-	-	+	-
#**AML 5**	46,XX<20>	IR	-	-	+	-	-	-	+
#**AML 6**	46,XX,t(1;16)(010;q10),inv(3)(014q24)<20>	HR							
#**AML 7**	46,XX<20>	IR	+	-	+	-	-	-	+
#**AML 8**	46,XY,r(7)<16>	HR							
#**AML 9**	46,XX<20>	IR	+	-	+	-	-	-	-
#**AML 10**	normal	IR	+	-	-	-	+	+	-
#**AML 11**	46,XY<22>	IR	-	+	+	-	-	-	-
#**AML 12**	46,XX<20>	IR	+	-	+	-	-	-	+
#**AML 13**	46,XY<20>	IR	+	-	-	-	-	-	-
#**AML 14**	normal (secondary)	IR	-	-	+	-	-	-	-
#**AML 15**	normal	IR	-	-	+	-	-	-	-
#**AML 16**	normal	IR	-	-	-	+	-	-	-
#**AML 17**	inv 16	LR							
#**AML 18**	normal	IR	-	-	+	-	-	-	-
#**AML 19**	inv 16	LR							
#**AML 20**	normal (secondary)	IR	+	-	-	-	-	-	-

n.d.: not determined.

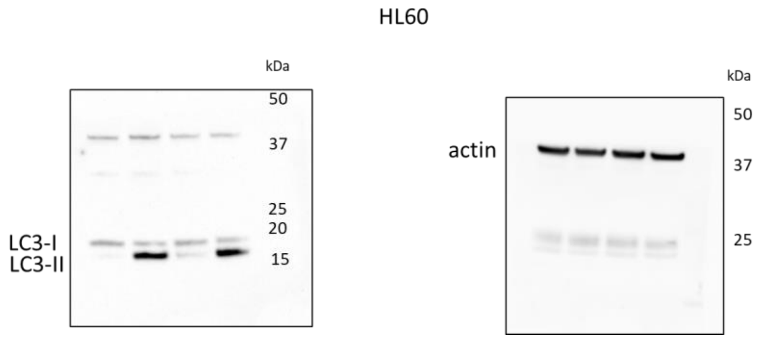

Uncropped Western blots from Figure 3B

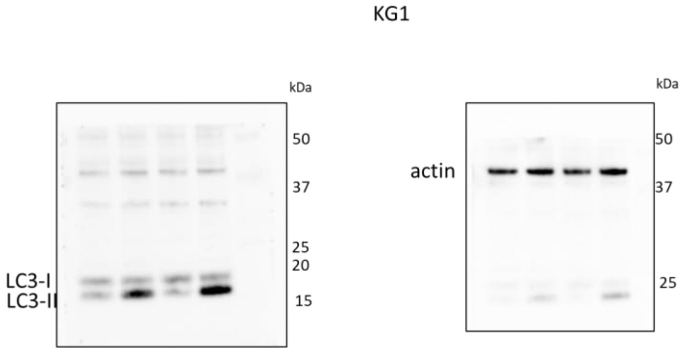

Western blots from Figure 3B

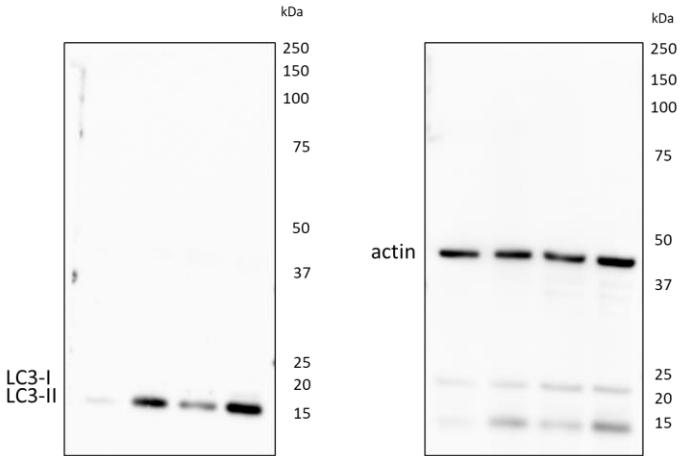

Western blots from Figure 5H

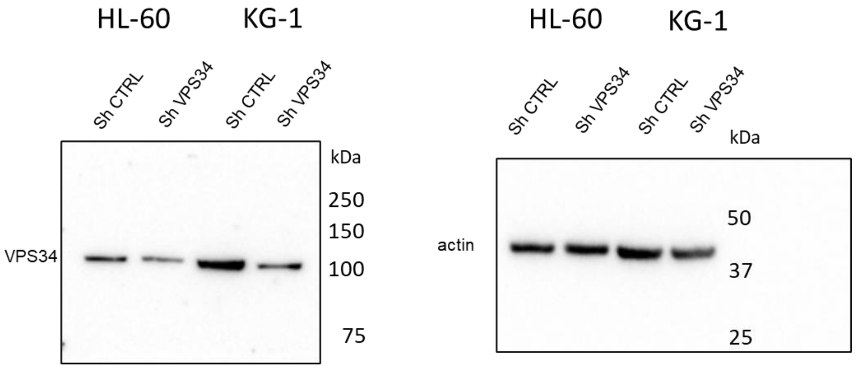

Western blots from supplementary Figure S1.

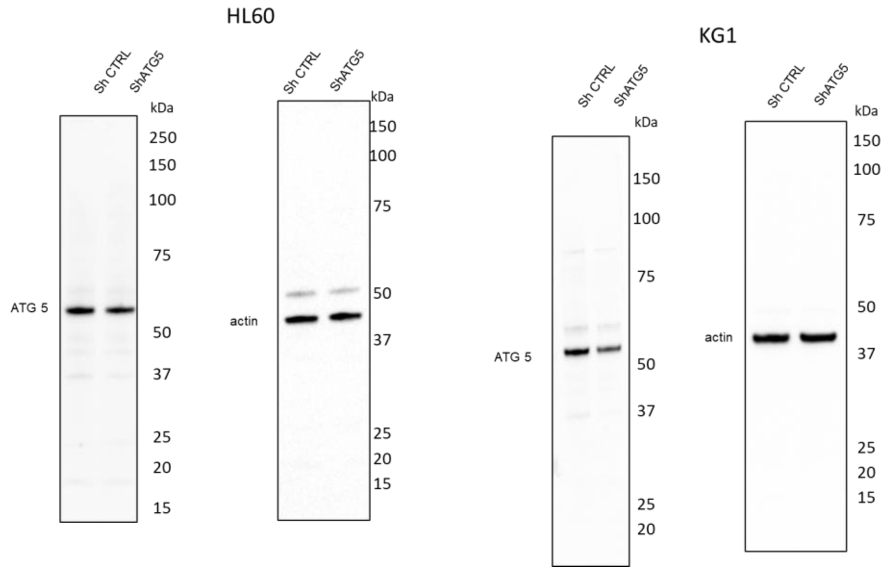

Western blots from supplementary Figure S1

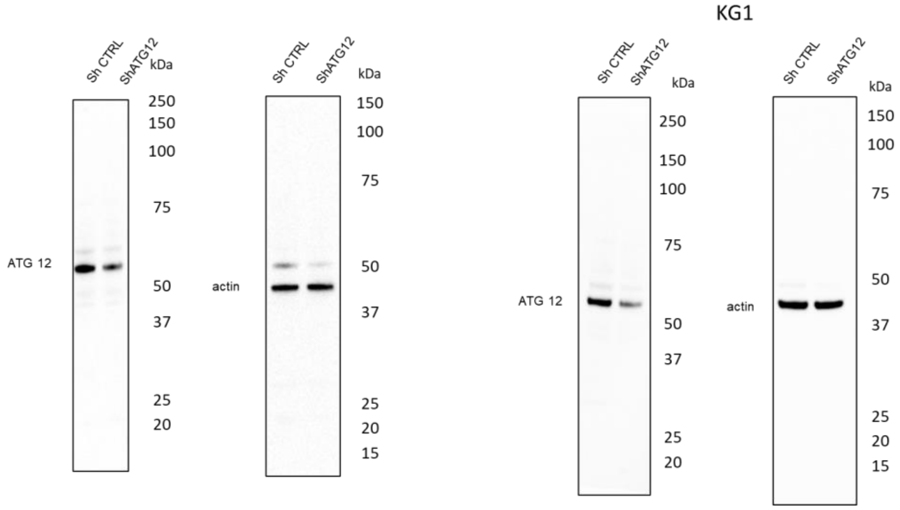

Western blots from supplementary Figure S1

Figure S3. Uncropped Western Blot Figures.

© 2020 by the authors. Licensee MDPI, Basel, Switzerland. This article is an open access article distributed under the terms and conditions of the Creative Commons Attribution (CC BY) license (http://creativecommons.org/licenses/by/4.0/).
